# Prognostic value of a five-lncRNA signature in esophageal squamous cell carcinoma

**DOI:** 10.1186/s12935-020-01480-9

**Published:** 2020-08-10

**Authors:** Lan zhang, Pan Li, Enjie Liu, Chenju Xing, Di Zhu, Jianying Zhang, Weiwei Wang, Guozhong Jiang

**Affiliations:** 1 Department of Pathology, The First Affiliated Hospital of Zhengzhou University, Zhengzhou University, Zhengzhou, 450052 Henan China; 2 State Key Laboratory of Esophageal Cancer Prevention & Treatment, Zhengzhou University, Zhengzhou, 450052 Henan China

**Keywords:** lncRNA, Esophageal squamous cell carcinoma, Overall survival, Signature, Prognosis

## Abstract

**Background:**

The aim of this study was to identify prognostic long non-coding RNAs (lncRNAs) and develop a multi-lncRNA signature for suvival prediction in esophageal squamous cell carcinoma (ESCC).

**Methods:**

The clinical and gene expression data from Gene Expression Omnibus database (GSE53624, n = 119) were obtianed as training set. A total of 98 paired ESCC tumor and normal tissues were detected by RNA sequencing and used as test set. Another 84 ESCC tissues were used for real-time quantitative PCR(qRT-PCR) and as an independent validation cohort. Survival analysis, Cox regression and Kaplan–Meier analysis were performed.

**Results:**

We screened a prognostic marker of ESCC from the GSE53624 dataset and named it as the five-lncRNA signature including AC007179.1, MORF4L2-AS1, RP11-488I20.9, RP13-30A9.2, RP4-735C1.6, which could classify patients into high- and low-risk groups with significantly different survival(median survival: 1.75 years vs. 4.01 years, log rank *P* < 0.05). Then test dataset and validation dataset confirmed that the five-lncRNA signature can determine the prognosis of ESCC patients. Predictive independence of the prognostic marker was proved by multivariable Cox regression analyses in the three datasets (*P* < 0.05). In addition, the signature was found to be better than TNM stage in terms of prognosis.

**Conclusion:**

The five-lncRNA signature could be a good prognostic biomarker for ESCC patients and has important clinical value.

## Background

Esophageal carcinoma is a malignant tumor of esophageal mucosa epithelium or gland. Globally, esophageal cancer ranks seventh in morbidity and sixth mortality among all cancers [1]. China is one of the countries with the highest incidence of esophageal cancer, accounting for approximately half of all esophageal cancer cases [2]. There were 477,900 new cases diagnosed in China in 2015 [3]. Among these Chinese esophageal cancer patients, esophageal squamous cell carcinoma (ESCC) patients account for nearly 90% [2]. In terms of mortality, esophageal cancer deaths in China account for half of global esophageal cancer deaths [4]. Therefore, ESCC is a major public health challenge in the world, especially in China. Using Chinese ESCC patients as research samples is of great significance in revealing the pathogenesis and prognostic factors of ESCC.

ESCC is an extremely aggressive malignancy. Along with the clinical symptoms of early esophageal squamous cell carcinoma are not obvious and can be easily ignored by patients, most ESCC patients are diagnosed in advanced stages. As a result, the prognosis of ESCC patients is extremely poor. The 5 year survival rate of ESCC patients in some less developed areas is less than 10% [5]. In the United States, where medical conditions are advanced, the 5 year survival rate is 18% [6]. In China, the 5 year relative survival of postoperative ESCC patients is 8%-41% [7, 8]. Despite recent advances in diagnostic and therapeutic approaches, the life expectancy of ESCC patients has not improved significantly [3, 9, 10]. In addition, the current global diagnosis of ESCC depends on endoscopy and biopsy [11]. After confirming the diagnosis, TNM stage is used clinically to make prognostic judgment and guide treatment, but the shortcoming of TNM stage is that it cannot achieve individualized prediction and accurate evaluation [12, 13]. Therefore, identification of a sensitive and effective prognostic marker of ESCC is in urgent need to evaluate disease progression and patient’s overall outcome.

Next generation sequencing (NGS) and bioinformatics analysis tools provides great help for human-beings to understand tumors [14–17]. In the process of exploring tumor pathogenesis and prognostic factors through NGS and bioinformatics analysis, long non-coding RNAs (lncRNAs), defined as transcripts longer than 200 bp, have been discovered to play important roles. For example, a HOX transcript antisense RNA, lncRNA HOTAIR, was found to correlate with poor prognosis of lung cancer and promote tumor progression [18]. LncRNA REG1CP was shown to promote the development and progression of tumor [19]. For ESCC, several lncRNAs have been detected to associate with short survival, such as lncRNA H19 [20], lncRNA CASC9 [21] and lncRNA LUCAT1 [22]. Subsequently, prognostic multi-lncRNA signatures with high potential clinical application significance were constructed based on lncRNA expression profile data from public database such as Gene Expression Omnibus (GEO) and The Cancer Genome Atlas (TCGA) database. For instance, a three-lncRNA signature (ENST00000435885.1, XLOC_013014, ENST00000547963.1) was found that it can classify the ESCC patients into two groups with significantly different overall survival [23]. A seven-lncRNA (RP5-1172N10.2, RP11-579D7.4, RP11-89N17.4, LA16c-325D7.2, RP1-251M9.2, RP11-259O2.2, LINC00173) signature can predict overall survival of ESCC patients [24]. Although prognostic lncRNA signatures have been identified for ESCC, there are few studies that can verify the effectiveness of the predictive model in independent experimental data set and confirm the value of the prognostic lncRNA in ESCC tissues.

Here, we used our lncRNA expression data tested by RNA sequencing and followed up the clinical 5 year survival information of 98 ESCC patients, then, combined it with 119 ESCC public expression profiles from GEO and another independent 84 ESCC tissues for qPCR validation to construct a clinically valuable lncRNA signature that can accurately predict survival of ESCC patients.

## Materials and methods

### Sample collection and preparation

LncRNA expression profile and corresponding clinical data of 119 ESCC cases (GSE53624) were obtained from the publicly available GEO database (https://www.ncbi.nlm.nih.gov/geo/). As Anyang is a high-risk area of ESCC in China, we collected 98 postoperative ESCC tissues and paired non-tumor tissues from Anyang Tumor Hospital during 2014–2015, and organized relevant patient clinical information. Then we examined the protein coding gene (PCG) and lncRNA expression profile of the 98 pair ESCC tissues by next-generation sequencing (NGS, Hereinafter referred to as RNA-seq dataset). In addition, we collected an independent validation cohort including 84 postoperative ESCC patients from the same hospital and detected their lncRNA expression level using the qRT-PCR. Detailed clinicopathological characteristics of all these ESCC patients of the ESCCs in this study was shown in Table 1. Tumor-node-metastasis (TNM) classification of the International Union against Cancer, 7th edition was used to categorize. Documentation of informed consent was obtained through the institutional review board. The study was approved by the Anyang Tumor Hospital Ethical Committee.

**Table 1:** Summary of ESCC patients and clinical characteristics in this study

Characteristic	GSE53624	RNA seq	qPCR validation
Group	Training	Test	Validation
Age (years)			
> 61	39	49	52
≤ 61	80	49	32
Sex			
Female	21	32	36
Male	98	66	48
Vital status			
Living	46	46	59
Dead	13	52	25
TNM stage			
Stage I	6	3	4
Stage II	47	51	51
Stage III	66	40	26
Stage IV	0	4	3

### RNA isolation and next generation RNA sequencing analysis

After TRIZOL lysis and purification, total RNA was isolated by the miRNeasy Mini Kit (QIAGEN) with DNase digestion step. A total amount of 5 ug RNA per sample was used as input material for the RNA sample preparation. Firstly, ribosomal RNA was removed by Epicentre Ribozero™ rRNA Removal Kit (Epicentre, USA), and rRNA free residue was cleaned up by ethanol precipitation. The sequencing libraries were generated by NEBNext Ultra™ Directional RNA Library Prep Kit for Illumina (NEB, USA) following manufacturer's recommendations and sequenced on an Illumina Hiseq platform. The 150 bp paired-end reads were generated. Normalized fragments per kilobase per million mapped reads (FPKM) by cufflinks was used to estimate the gene expression values.

### Validation of lncRNA expression using RT-PCR

LncRNAs reverse transcription were amplified with TIANScript II RT Kit (KR107, TIANGEN, Beijing, China). We used real-time quantitative PCR (qRT-PCR) to measure the lncRNA expression with TB Green® Premix Ex Taq™ (Tli RNaseH Plus, TaKaRa, Dalian, China). Relative quantification of lncRNA expression was normalized by the 2^−ΔCt^ method, and GAPDH was used for normalization with the corresponding primers (Additional file 1. Table S1). All reactions were carried out in triplicate by StepOnePlus™ Real-Time PCR System (Applied Biosystems) [25–27].

### Construction of multi-lncRNA prognostic signature

GSE53624 and RNA-seq datasets were analyzed respectively by Cox analysis and Kaplan–Meier analysis to identify lncRNAs associated with overall survival (OS) of ESCC patients. We selected the prognostic lncRNAs using Cox p & log rank *P* < 0.05 in 2 datasets. Model was estimated as follows [25, 26, 28]: Risk Score (RS) = ∑ ^N^_i=1_ (*ExpLncRNA*_i_ * *CoefCox*_i_), where N is prognostic lncRNA number, *ExpLncRNA*_i_ represents the lncRNA expression value, and *CoefCox*_i_ is the Cox regression coefficient of lncRNA. We plotted ROC curves [16] and calculated their area under the curve (AUC) values to screen out the prognostic signature with the largest AUC value in the GSE53624 set [28]. The whole construction process was shown in Additional file 2. Figure S1. R program (Version 3.5.1) was used to perform the above analyses, including packages called pROC, TimeROC and survival from Bio-conductor (https://www.bioconductor.org/). SubpathwayMiner was used to find the selected prognostic genes related pathways (https://cran.r-project.org/web/packages/SubpathwayMiner/), which provides more flexibility in annotating gene sets and identifying the involved pathways (entire pathways and sub-pathways) [29].

## Results

### Screening prognostic lncRNAs in ESCC

**Fig. 1:**
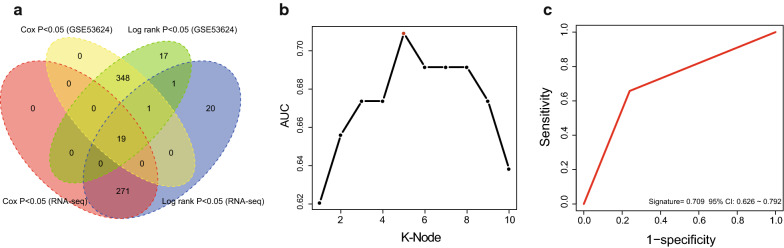
Constructing the prognostic lncRNA model in the high throughput sequencing datasets. **a** Venn diagram for analyzing the prognostic lncRNAs. **b** Screening out the lncRNA signature with largest AUC from all 1023 signatures which were calculated by ROC for k = 1, 2…… 10. **c** The AUC of the screened lncRNA signature

There were a total of 217 samples with lncRNA expression profiles including GSE53624 (n = 119) and RNA-seq (n = 98) dataset in this study. From the above two datasets, we found a total of 6253 lncRNAs were expressed in ESCC tissues. We then performed univariate cox analysis and Kaplan–Meier analysis to analyze the relationship between the lncRNA expression and ESCC OS. Based on the two ESCC profiling datasets and the corresponding clinical follow-up information, univariate cox analyses identified 368 lncRNAs in the GSE53624 and 290 lncRNAs in the RNA-seq dataset which were significantly associated with ESCC OS (Cox *P* < 0.05) and Kaplan–Meier analyses identified 386 survival associated lncRNAs in the GSE53624 and 312 survival associated lncRNAs in the RNA-seq dataset (log rank *P* < 0.05). Through comparing the above four groups of survival related lncRNA data, we found 19 identical lncRNAs significantly correlated with OS in two datasets (COX *P* < 0.05, log rank *P* < 0.05) (Fig. 1a). Subsequently, we found that 10 of the 19 prognostic-related lncRNAs showed a consistent risk trend in the four groups, displaying a risk or protective role in ESCC (Additional file 3. Table S2).

### Constructing prognostic multi-lncRNA signatures

We used 119 ESCC data from public GSE53624 as the training group, 98 RNA-seq data as test group and 84 qPCR data as validation group. Using above selected 10 prognostic lncRNAs, we constructed 2^10^–1 = 1023 signatures in the training dataset. In order to obtain a signature with the strongest prognostic ability, we performed ROC analyses in the training dataset (Additional file 4. Table S3). We screened the 5-lncRNA combination with the largest AUC value, namely the prognostic signature containing AC007179.1, MORF4L2-AS1, RP11-488I20.9, RP13-30A9.2, RP4-735C1.6 (Fig. 1b, Table 2). The lncRNA risk score was calculated as follows: (0.47 × expression value of AC007179.1) + (0.58 × expression value of MORF4L2-AS1) + (-0.47 × expression value of RP11-488I20.9) + (-0.64 × expression value of RP13-30A9.2) + (0.62 × expression value of RP4-735C1.6). As shown in Fig. 1c, the AUC of the screened lncRNA signature was 0.71.

**Table 2:** The selected lncRNAs in the prognostic signature of ESCC

Gene symbol	Coefficient^a^	*P* value^a^	*KM* *P* value	Expression level association with prognosis
RP13-30A9.2	− 0.635	0.008	0.007	Low
RP11-488I20.9	− 0.468	0.048	0.046	Low
MORF4L2-AS1	0.582	0.014	0.013	High
AC007179.1	0.470	0.047	0.045	High
RP4-735C1.6	0.616	0.010	0.008	High

^a^Derived from the univariable Cox regression analysis in the GSE53624 set; *KM* Kaplan–Meier analysis

### Predictive power of the lncRNA signature for patients with ESCC

**Fig. 2:**
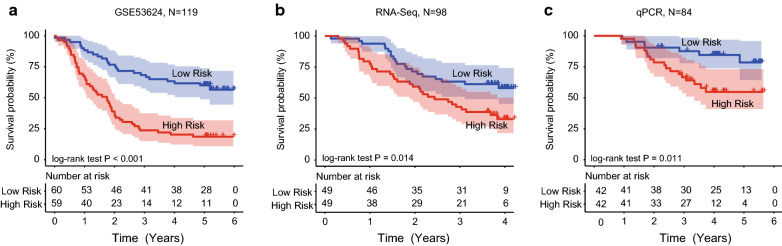
The lncRNA signature classification power for ESCC prognosis. Kaplan–Meier curves found ESCC patients were classified into two different risk groups based on the risk score of the signature in the GSE53624 (**a**), RNA-seq (**b**) and qPCR validation datasets (**c**)

After calculated risk scores of ESCC patients in the GSE53624 dataset, the median risk classified ESCC patients into high- and low- risk groups (n = 59/60). Kaplan–Meier analysis found the survival of ESCC patients in the low-risk group was significantly longer than those in the high-risk group (median survival: 1.75 years vs. 4.01 years, log-rank test *P* < 0.001; Fig. 2a). The 3 year survival rate for patients in the high-risk group was only 23.73%, while that of patients in the low-risk group reached as high as 66.67%.

Then we evaluated the predictive ability of the five-lncRNA signature in ESCC RNA-seq dataset (n = 98), which was treated as the test set. The median risk score of patients in the test dataset was calculated and used to divide patients into high- and low-risk groups. Figure 2b showed the Kaplan–Meier analysis result. The prognosis of ESCC patients in the low-risk group was significantly better than patients in the high-risk group (Median survival: 2.44 years vs. 3.97 years, log-rank test *P* = 0.014; Fig. 2b). The 3 year survival rate for the patients of high-risk group was only 40.82%, while that of the low-risk group was 61.22%.

In addition, we performed qPCR experiment and obtained the five lncRNAs expression level in 84 ESCC tissues. Through calculated risk scores and used the median risk score as the cutoff value, we obtained the high score group and low score group. Kaplan–Meier analysis discovered the survival difference between two risk groups. The survival of ESCC patients with low-risk scores was still significantly greater than patients with high-risk scores. (Median survival: 2.82 years vs. 4.08 years, log-rank test *P* = 0.011; Fig. 2c).

**Fig. 3:**
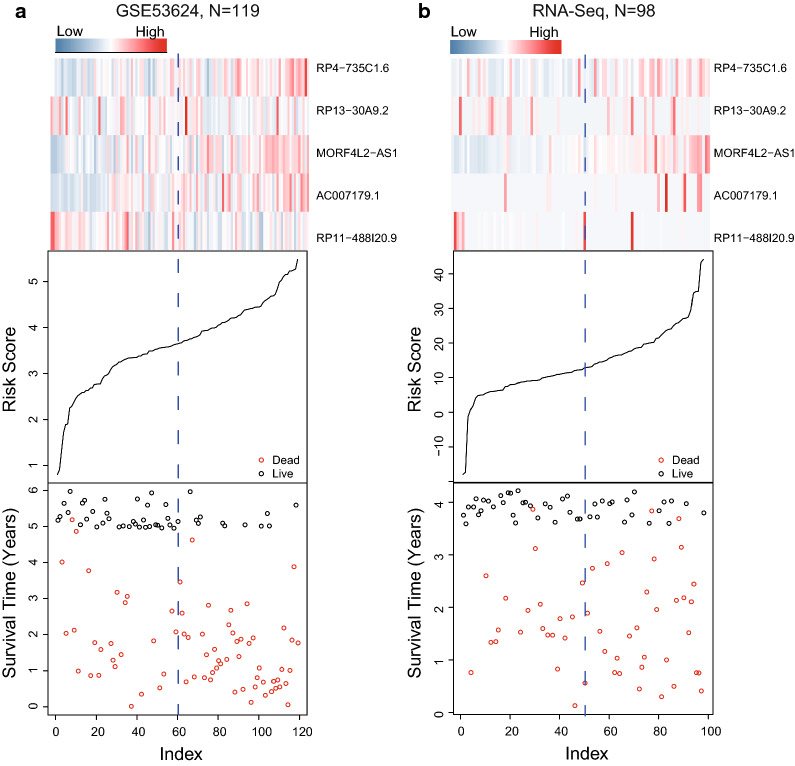
Risk score distribution, survival status and gene expression of ESCC patients in high- and low-risk groups classified by the four-lncRNA signature in the GSE53624 (**a**), RNA-seq (**b**) datasets

Figure 3 displayed the survival time, risk score and lncRNA expression level of each patient in above two ESCC lncRNA expression profile datasets, including GSE53624 and RNA-seq datasets. As shown, patients with the higher expression of AC007179.1, MORF4L2-AS1 and RP4-735C1.6, the higher the risk were with the more deaths. On the contrary, patients with lower-risk scores tended to have higher expression level of protective lncRNAs (RP13-30A9.2, RP11-488I20.9) and have a lower mortality rate.

### The five-lncRNA signature can independently predict the survival of ESCC patients

The Chi-square test found the lncRNA signature was not associated with clinicopathological factors, including age, sex and pTNM stage in the training, test and qPCR validation groups (Table 3). Although univariate cox analysis of the training, test and independent validation datasets showed TNM stage and the signature were both associated with the prognosis of ESCC patients, multivariable Cox regression analyses showed only the lncRNA signature was an independent prognostic factor for ESCC patients (High-risk group vs. Low-risk group, HR training = 3.50, 95% CI 2.13–5.76, *P* < 0.001, n = 119; HR test = 1.03, 95% CI 1.00–1.06, *P* = 0.014, n = 98; HR independent = 2.95, 95% CI 1.21–7.17, *P* = 0.017, n = 84, Table 4).

**Table 3:** Association of the signature with clinicopathological characteristics in ESCC patients

Variables	GSE53624		*P*	RNA-seq		*P*	qPCR		*P*
	Low*	High*		Low*	Hig *		Low*	High*	
Age			0.217			0.106			0.261
≤ 62	44	36		29	20		13	19	
> 62	16	23		20	29		29	23	
Sex			> 0.99			0.132			0.825
Female	11	10		20	12		17	19	
Male	49	49		29	37		25	23	
TNM stage			0.654			0.588			0.681
Stage I	2	4		1	2		3	1	
Stage II	25	22		28	23		25	26	
Stage III	33	33		19	21		12	14	
Stage IV				1	3		2	1	

*Low risk ≤ Median of risk score, High risk > Median of risk score; The Chi-squared test *P* value < 0.05 was considered significant

**Table 4:** Cox regression analysis of the signature for the ESCC patients from the three datasets

		Univariable analysis				Multivariable analysis			
Variables		HR	95% CI of HR		*P*	HR	95% CI of HR		*P*
			Lower	Upper			Lower	Upper	
GSE53624 dataset(n = 119)									
Age	> 61 vs. ≤ 61	1.643	1.024	2.636	0.040	1.441	0.894	2.322	0.134
Sex	Male vs. Female	0.827	0.468	1.461	0.513	0.782	0.440	1.391	0.403
TNM stage	III, IV vs. I, II	1.901	1.226	2.948	0.004	2.017	1.288	3.159	0.002
lncRNA signature	High risk vs. low risk	3.264	1.998	5.331	< 0.001	3.502	2.131	5.756	< 0.001
RNA seq set (n = 98)									
Age	> 61 vs. ≤ 61	1.287	0.744	2.225	0.367	1.314	0.711	2.430	0.383
Sex	Male vs. Female	1.534	0.818	2.875	0.182	1.116	0.561	2.221	0.754
TNM stage	III, IV vs. I, II	1.795	1.152	2.796	0.010	1.629	0.887	2.991	0.115
lncRNA signature	High risk vs. low risk	1.994	1.139	3.491	0.016	1.032	1.002	1.062	0.035
Independent qPCR validation set (n = 84)									
Age	> 61 vs. ≤ 61	0.949	0.892	1.011	0.105	0.961	0.893	1.034	0.289
Sex	Male vs. Female	0.797	0.352	1.804	0.585	0.872	0.381	1.997	0.747
TNM stage	III, IV vs. I, II	2.699	1.607	4.534	0.000	4.910	2.133	11.302	< 0.001
lncRNA signature	High risk vs. low risk	2.981	1.235	7.192	0.015	2.945	1.210	7.168	0.017

### The prediction performance of the five-lncRNA signature and TNM stage

**Fig. 4:**
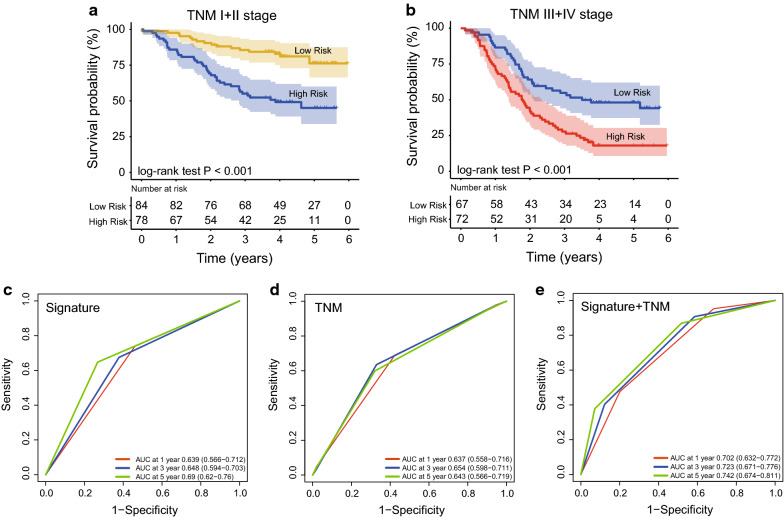
Stratification analysis of TNM stage using the five-lncRNA signature. **a**, **b** Stratification analysis of TNM low/high stage using the signature by Kaplan–Meier curves. **c**, **d** Comparing the survival prediction power of the signature with that of TNM stage by ROC in the entire datasets. **e** TimeROC analysis to explore the survival prediction power of combination of the signature and TNM stage in the entire dataset

TNM stage is currently used as the main indicator for prognosis evaluation of ESCC. Our study confirmed that TNM stage and the five-lncRNA signature were related to the prognosis of patients. Therefore, we performed stratification analysis to explore the predictive power of the five-lncRNA signature in patients with known TNM stage. We first divided all the ESCC patients involved in this study (n = 301) into 2 groups, one group was patients with TNM low (I + II) stage and the other group was patients with high TNM stage (III + IV). Then we used the lncRNA signature to conduct stratification analyses of above two groups. Kaplan–Meier results showed that the lncRNA signature could further separate the patients with TNM low (I + II) stage (Fig. 4a) or patients with high TNM stage (III + IV)(Fig. 4b) into two subgroups with significantly different survival(log-rank test P < 0.001).

To analyze the predictive performance advantages of the five-lncRNA signature, TimeROC analyses were performed in the three datasets (n = 301). The AUCs of the signature were 0.690/0.648/0.639 at 5/3/1 years (Fig. 4c), while the AUCs of TNM were 0.643/0.654/0.637 at 5/3/1 years (Fig. 4d), suggesting the five-lncRNA signature was superior to TNM stage in terms of ESCC prognosis evaluation, especially at 5 years survival. Moreover, we found AUC values were maximized when signature and TNM staging were combined to assess patient prognosis (0.742/0.723/0.702 at 5/3/1 years, Fig. 4e), indicating the lncRNA signature improve the accuracy of TNM in evaluating prognosis.

### Functional prediction of the five-lncRNAs signature

**Fig. 5:**
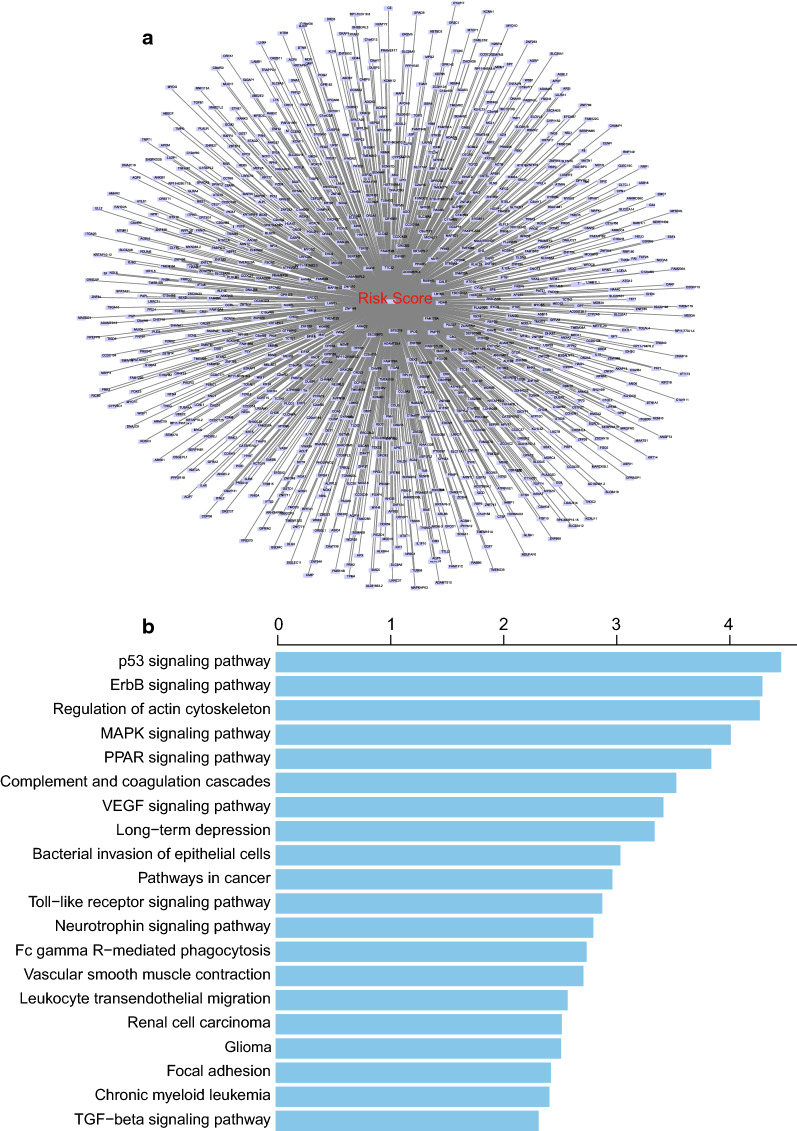
Functional prediction of the five-lncRNAs signature. Visualization of the co-expressing genes with the risk score signature in GS53624 and RNA-Seq datasets (**a**). Functional enrichment analysis of those co-expressing genes by SubpathwayMiner (**b**)

In order to predict function of the five-lncRNAs signature, we performed Pearson analysis to obtain the genes implicated in the correlation of these 5-lncRNA signature in the GSE53624 and RNAseq datasets, respectively. Then we got 988 co-expression genes in total (Pearson coefficient > 0.4/ < -0.4, P < 0.001, Fig. 5a). SubpathwayMiner suggested these 988 genes were significantly enriched in 73 different KEGG pathways (*P* < 0.05, Additional file 5. Table S4), especially involved in p53 signaling pathway, ErbB signaling pathway, Regulation of actin cytoskeleton, MAPK signaling pathway, PPAR signaling pathway VEGF signaling pathway, Pathways in cancer and Toll-like receptor signaling pathway (Fig. 5b), which were ranked in the top 20 and were closely related to the cancer development.

## Discussion

Esophageal squamous cell carcinoma is a rapidly progressive disease faced with many difficulties, such as difficulty in early diagnosis, low 5 year survival rate, and lack of effective prognostic markers. Recently, lncRNA has been reported to be involved in the occurrence and progression of tumors [30, 31]. However, the expression characteristics and roles of lncRNAs in ESCC are still fairly elusive. Thus, this paper revealed the survival related lncRNAs expression level in ESCC and constructed a prognostic five-lncRNA signature by collected public ESCC lncRNA expression data. Then, we measured the lncRNA expression of 98 ESCC patients by RNA seq and test the lncRNA expression of 84 ESCC tissues for validating the predictive performance of the five-lncRNA signature. Meanwhile, the 98 ESCC lncRNA expression profiles provide scientists with reference data to study the association of ESCC and lncRNAs.

Despite numerous research have reported some ESCC lncRNA models for prognostic markers [24], PCR validation was rarely performed in the independent validation group and some lncRNAs in the prediction model might not yet exist in ESCC tissue. In this study, the predictive model was constructed through the analyzing two sets of high-throughput sequencing data, and lncRNAs of the five-lncRNA signature were shown to be present in collected ESCC samples by qPCR.

In the five-lncRNA suvival prediction model, the high expression level of lncRNA MORF4L2-AS1, AC007179.1 and RP4-735C1.6 were associated with short survival (Cox coefficient > 0), indicating these three lncRNAs were risk lncRNAs for ESCC patients. The high expression of RP13-30A9.2 and RP11-488I20.9 were associated with long survival (Cox coefficient < 0), suggesting the two lncRNAs were protective lncRNAs for ESCC. The significant correlation of the five lncRNAs with ESCC prognosis was found in two data sets, emphasizing the important clinical research value of the five prognostic lncRNAs in ESCC. However, the function of prognostic lncRNAs in ESCC have not been reported so far and biological roles of the five lncRNAs in ESCC progression should be investigated in further experimental studies.

TNM stage is a commonly used tumor classification standard in clinical practice and a recognized prognostic marker [32, 33]. However, TNM stage remains flawed in prognostic assessment. We found that the prognostic predictive power of signature is better than TNM stage, suggesting that the strong prognostic ability of the five-lncRNA signature. Consistent with some scholars’ results that combined TNM classification with molecular marker can predict outcome of ESCC patients more accurately [34], we found the prognostic prediction of the combination of signature with TNM stage was the best, indicating the signature combined with TNM stageg is useful for prognosis evaluation.

## Conclusion

We identified a prognostic five-lncRNA signature from a large cohort of ESCC patients. The signature could predict the survival of patients with ESCC based on lncRNA expression profile and have strong clinical value.

## Supplementary information

**Additional file 1: Table S1.** The detail of lncRNAs and GAPDH primers in this study.

**Additional file 2: Figure S1.** The schedule of analyses to construct the lncRNA signature in this study.

**Additional file 3: Table S2.** Ten out of the 19 prognostic-related lncRNAs showed a consistent risk trend in the high throughput sequencing data.

**Additional file 4: Table S3.** The 1023 signatures comprising lncRNAs in the GSE53624 dataset (n = 119)

**Additional file 5: Table S4.** Functional enrichment of those co-expressed genes with the risk score by SubpathwayMiner analysis.

## Data Availability

The data generated during this study are available from the corresponding author upon reasonable request. The data that support the findings of this study are available in Gene Expression Omnibus database (GEO). These data were derived from the following resources available in the public domain: https://www.ncbi.nlm.nih.gov/geo/query/acc.cgi?acc=GSE53624.

## Abbreviations

ESCC: Esophageal squamous cell carcinoma
qPCR: Real-time quantitative PCR
ROC: Receiver operating characteristic
AUC: Area under the ROC curve
CI: Confidence interval
OS: Overall survival
