# Supplementary figures and images for: Prognostic value of a five-lncRNA signature in esophageal squamous cell carcinoma
Source: Cancer Cell Int. 2020 Aug 10;20:386. doi: 10.1186/s12935-020-01480-9 (PMC7419219; doi:10.1186/s12935-020-01480-9)

# Figure S1

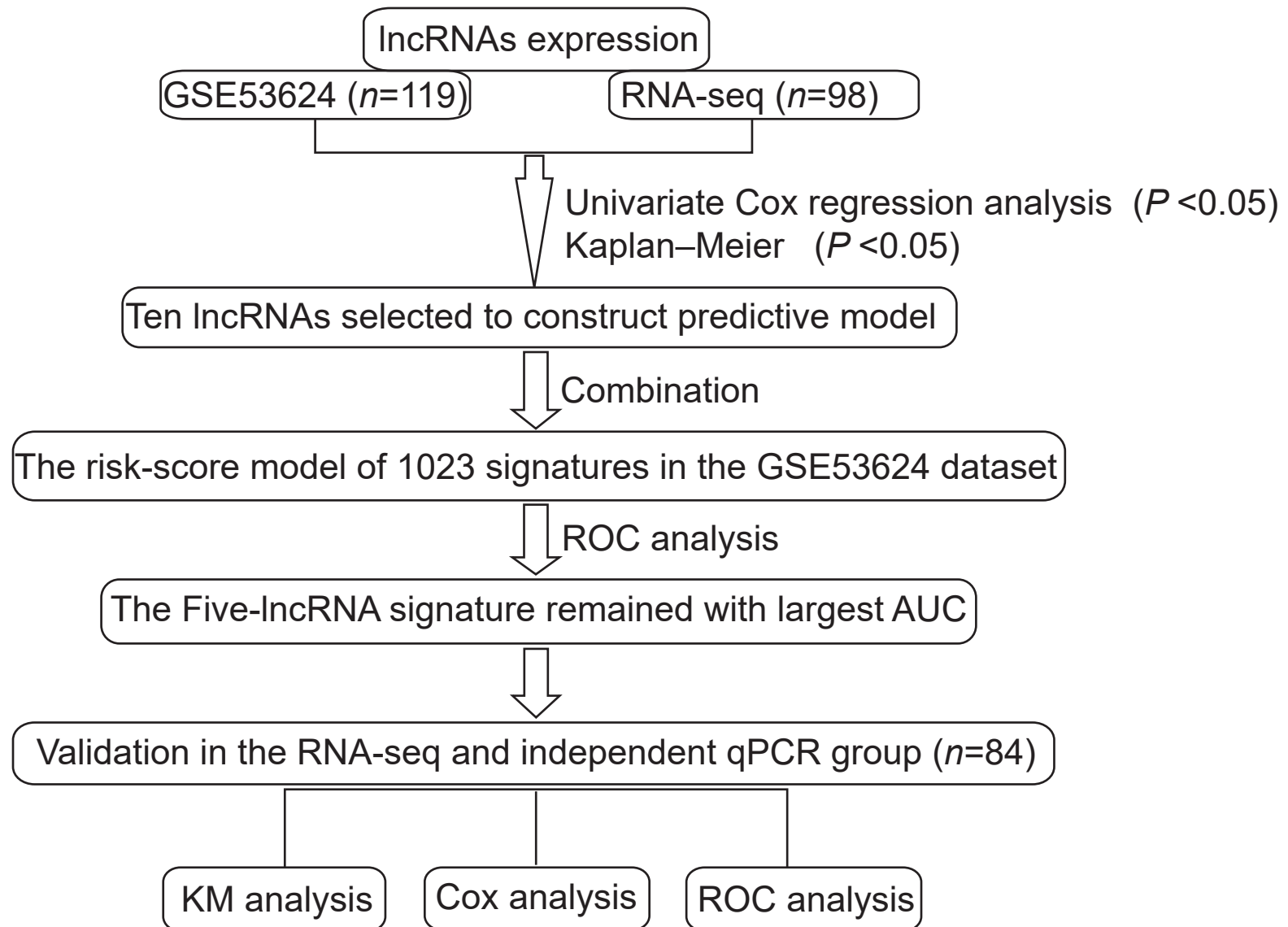

Supplement: Supplementary file 2 — Additional file 2: Figure S1. The schedule of analyses to construct the lncRNA signature in this study. [file 12935_2020_1480_MOESM2_ESM.pdf]
